# Reflex Conditions in Gross Injuries of the Spinal Cord

**Published:** 1918-08

**Authors:** 


***The Automatic Bladder, Excessive Sweating, and Other Reflex Conditions in Gross Injuries of the Spinal Cord.*** By Henry Head and Geo. Riddoch. *Brain,* Vol. XL, pts. 2 and 3.

This extremely important article has opened up a new field not only in the conception of bladder disturbances following spinal cord injury but, what is more important, in the *treatment* of these disturbances. The monograph is divided into three chapters and an appendix which includes illustrative cases. These conclusions are so all-inclusive of the contents of the article that a summary of them will contain all the important results.

(A) The Automatic Bladder. (1) When the spinal cord has been completely divided, the bladder may begin under favorable conditions to expel its contents automatically as early as twenty-five days after the injury.

In unfavorable cases, where the patient suffers from chronic septicemia, due to a bed-sore, grave cystitis, or pyelitis, automatic micturition may never become established, however long the patient survives the injury.

(2) If a catheter is passed after automatic but involuntary micturition has become established, and fluid is allowed to run into the bladder under the least possible pressure, it will be expelled after a certain volume has entered. This volume varies between about 100 and 600 cc.

This expulsion of fluid through a catheter, owing to a contraction of the muscles of the bladder wall, can be evoked at a time when the patient is unable to micturate automatically, that is to say, during the period of complete retention. This retention is due to spasmodic contraction of the sphincter mechanism, which does not relax although the muscular wall of the bladder is capable of vigorous contraction.

Later, under favorable general conditions, true automatic micturition may become established, even although the spinal cord is completely divided. When the contents of the bladder reach a certain amount, its muscular wall contracts, the sphincter relaxes, and urine is passed involuntarily.

(3) The form assumed by the activity of such an automatic bladder is entirely independent of the site of the lesion in the spinal cord.

(4) If the spinal cord is gravely injured and if it is even completely divided anywhere above the lumbar region, automatic evacuation and the complete act of micturition may be facilitated by the most afferent impulses passing into the lower portion of the spinal cord.

When the sole of the foot, the thigh, or the abdomen is scratched a flexor spasm may be evoked. If this is so, the bladder may empty itself when its contents scarcely reach one-half of the amount otherwise necessary to produce a contraction of the muscular wall.

(5) After destruction of the lower lumbar and sacral roots, the bladder may act automatically in an identical manner, except that it can no longer be influenced reflexly by any afferent impulses.

(6) When the lesion is confined to the lower end of the spinal cord or to the lower lumbar and sacral nerve roots, the patient may be conscious of tension within his bladder. may recognize the occurrence of contraction in its muscular wall, and may experience that pleasure which normally accompanies evacuation. But these sensations have no effect on the automatic activity of the bladder.

(7) When the bladder is acting automatically, deep breathing may cause the muscular wall to expel its contents before they have reached sufficient volume to be otherwise an adequate stimulus.

In the same way, pressure on the abdominal wall not only tends to expel the contents of the bladder mechanically, but also acts as stimulus to otherwise premature evacuation.

(8) When washing out a bladder, in order to treat the cystitis so frequently present in patients with spinal lesions, it is most important to avoid undue tension on the bladder wall. The fluid should be allowed to run in under the smallest pressure; the vessel containing the wash must not be raised, as is so commonly done, some feet above the bed, nor must the bladder be injected from a syringe. Such methods are positively harmful; for the tone and contractile capacity of the vesical musculature are overcome by such pressure, and the power of spontaneous evacuation thereby diminished. In every case where an automatic bladder is to be washed out for therapeutic purposes, the volume of fluid should be determined at which evacuation occurs, and the bladder should always be allowed to empty itself, as far as possible, in response to endo-vesical stimuli.

*Excessive Sweating* : (9). In most cases of hyperidrosis, associated with gross lesions of the spinal cord, the outburst of sweating represents the activity of the nervous system *below* the lesions.

Thus when the injury is situated in the lower cervical segments, the whole head and neck may be covered with sweat, because all the fibers which produce sweating leave the spinal cord in the thoracico-lumbar region. A lesion at the level of the third thoracic segment caused excessive sweating over both arms and over the trunk below the second rib; in a case where the injury lay in the sixth thoracic segment, hyperidrosis extended from the fifth rib downwards with some moisture of the palms of the hands. When the injury is situated at the ninth thoracic segment, the hyperidrosis corresponded almost exactly with the analgesia, whilst below this segment the area occupied by this sweating was smaller than that of the loss of sensation. In some cases this sweating may be unilateral.

(10) In favorable cases paroxysmal sweating can be excited by almost any stimulus which sends afferent impulses into the spinal cord below the lesion, for instance : —

*a*) scratching the sole of the foot, the abdomen, or any part below the level of the lesion, provided it evokes flexor spasms.

*b*) injection of fluid into the bladder. Inability to pass urine when the bladder is full, is one of the most frequent causes of " spontaneous " hyperidrosis in these cases; sweating ceases when a catheter is passed and the endo-vesical pressure is relieved.

(*c*) injection of fluid into the lower bowel, as when administering an enema.

*Reflex Activity of the Spinal Cord below the Lesion* : (11) It is evident therefore that under certain conditions the spinal cord below the level of the lesion may show signs of diffuse reflex activity. Scratching the sole of the foot may not only evoke a flexor spasm, but may cause premature evacuation of the bladder and an outburst of excessive sweating. This we have spoken of as a " mass-reflex ".

When the spinal cord has assumed this form of activity, any stimulus giving rise to impulses endowed with affective tone will evoke this diffuse reaction. Not only can evacuation of the bladder and rectum be facilitated by scratching the sole of the foot, but injecting fluid into either of these organs may evoke characteristic flexor spasms.

(12) When the spinal cord reacts with this massive response, it is obvious that the reflexes have, to a great extent, lost their local signature. Not only is the answer the same when diverse parts, such as the sole of the foot, the bladder, or the rectum are stimulated, but it matters little what part of the skin is scratched provided it is supplied by the spinal cord below the lesion. Scratching the abdomen or the sole of the foot may evoke the same reflex response.

(13) Should the local sign of the reflexes be retained to such an extent that primary extension can be evoked by scratching the thigh, though flexion is caused by nociseptive stimuli to the sole of the foot, this diffuse and massive response fails to appear. Evacuation of the bladder cannot be facilitated from the limb which yields a primary extensor response to stimulation of the thigh. We believe that the presence of reflex stepping is another indication that the reflexes have not entirely lost their local signature.

(14) All voluntary movement, sensation, and visceral control may be lost below the level of the lesion, and yet excessive facilitation may not be present. This may be due to the following causes : —

*a*) The patient may suffer from continuous septicemia or toxic absorption, due to a foul bed-sore, cystitis, or pyelitis. The lower end of the spinal cord has no chance to recover from the first period of shock due to the injury, and reflex activity does not return to any material extent, however long the patient may live.

*b*) In spite of the severity of the injury, the centers above the level of the lesion may still exercise sufficient control to prevent this widespread reflex activity. The severity of the injury has not been sufficiently great to prevent the neural mechanism for postural adaptation from influencing the parts below the lesion, though voluntary motion, sensation, and visceral control may be lost. The maintenance of postural activity in man is shown by the existence of primary extensor reactions and the presence of local signature which can be obtained from parts below the lesion.

